# Nano-Biofertilizer Formulations for Agriculture: A Systematic Review on Recent Advances and Prospective Applications

**DOI:** 10.3390/bioengineering10091010

**Published:** 2023-08-25

**Authors:** Diksha Garg, Kandi Sridhar, Baskaran Stephen Inbaraj, Prince Chawla, Manikant Tripathi, Minaxi Sharma

**Affiliations:** 1 Department of Microbiology, Punjab Agricultural University, Ludhiana 141004, India; 2 Department of Food Technology, Karpagam Academy of Higher Education (Deemed to be University), Coimbatore 641021, India; 3 Department of Food Science, Fu Jen Catholic University, New Taipei City 242062, Taiwan; 4 Department of Food Technology and Nutrition, Lovely Professional University, Phagwara 144411, India; 5 Biotechnology Program, Dr. Rammanohar Lohia Avadh University, Ayodhya 224001, India; 6 CARAH ASBL, Rue Paul Pastur, 11, 7800 Ath, Belgium

**Keywords:** nano-biofertilizer formulations, encapsulation, chitosan, polysaccharide, microorganism, plant growth promotion, smart farming

## Abstract

In the twenty-first century, nanotechnology has emerged as a potentially game-changing innovation. Essential minerals are mostly unavailable in modern cropping systems without the application of synthetic fertilizers, which have a serious negative impact on the ecosystem. This review focuses on the coupling of nanoparticles with biofertilizers to function as nano-biofertilizers (NBFs), which may ensure world food security in the face of the rising population. The inoculation of plants with NBFs improves plant development and resistance to stress. Metallic nanoparticles as well as organic components comprising polysaccharide and chitosan may be encapsulated, utilizing microbe-based green synthesis to make NBFs, which circumvents the limitations of conventional chemical fertilizers. The application of NBFs is just getting started, and shows more promise than other approaches for changing conventional farming into high-tech “smart” farming. This study used bibliographic analysis using Web of Science to find relevant papers on “nano biofertilizers”, “plants”, and “agriculture”. These subjects have received a lot of attention in the literature, as shown by the co-citation patterns of these publications. The novel use of nanotechnology in agriculture is explored in this research work, which makes use of the unique characteristics of nanoscale materials to address urgent concerns including nutrient delivery, crop protection, and sustainable farming methods. This study attempts to fill in some of the gaps in our knowledge by discussing the formulation, fabrication, and characterization of NBFs, as well as elucidating the mechanisms by which NBFs interact with plants and how this benefits the ability of the plant to withstand biotic and abiotic stress brought about by climate change. This review also addresses recent developments and future directions in farming using NBF formulations in the field.

## 1. Introduction

In the last ten years, the emergence of nanotechnology as a game-changing technology has spread throughout the life sciences. It is possible to enter the realm of nanotechnology through the study of nanoparticles (NPs), which are materials with very small particle sizes, typically between 1 and 100 nm. Particles in the nanoscale have unique optical, magnetic, and electrochemical properties that depend on their size [1]. Nanotechnology, which is based on the varying sizes of nanoparticles, is among the fastest-growing fields of research in material science right now. The scope of nanotechnology has widened considerably since its inception [2]. The use and application of nanotechnology have grown as it has been increasingly combined with other scientific disciplines such as chemistry, biology, physics, agriculture, etc. Combining nanotechnology with biotechnology has greatly expanded the field’s potential applications [3]. In many third-world nations, agriculture is the main source of income for the population [4]. Indirectly or directly, it is one of the most important industries, since it provides food, fiber, fuel, and heat to humans. Problems have arisen for the agricultural sector worldwide due to factors such as global warming, increased urbanization, and the careless application of agrochemicals. These problems are made worse by a growing population, with correspondingly higher food demand [5], which further calls for greater agricultural yield using environmentally sustainable methods. Supporting rural populations, cutting food and agri-waste, increasing the capabilities of irrigation methods, and addressing soil degradation are just a few of the many social and economic measures that must be taken to reach the goal of food security. 

**Figure 1:**
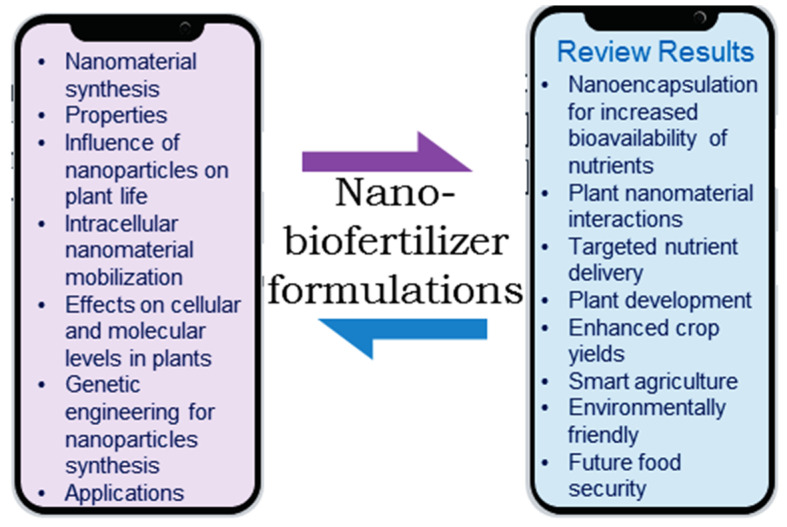
Schematic representation of the review.

Without a doubt, advancements in science and technology will play a crucial role in boosting agricultural output. As an exciting new branch of science, nanotechnology allows for cutting-edge investigations in the agriculture sector by exploiting revolutionary physical and chemical characteristics of the material at the nanoscale. It has the potential to be developed as a viable answer to existing agricultural difficulties, and it is a useful instrument for stimulating a new era of precision farming practices [6]. The application of nanotechnology in crops is promising. It is widely documented that optimal crop production can only be achieved by providing plants with the sixteen nutrients—both macro- and micronutrients—that they require [7]. Both macro- and micronutrients can be turned into nanoscale fertilizers, but nano-phosphorus (NP) and nano-nitrogen (NN) fertilizers will be particularly useful due to the significant demand for these nutrients among vegetable crops. These fertilizers will resolve the fundamental issues with traditional micronutrient fertilizers. This review addresses the transformative potential of nanotechnology in the agricultural industry. More specifically, the research exploits the one-of-a-kind physical and chemical properties of nanoscale materials in order to address urgent issues such as the delivery of nutrients, the protection of crops, and the implementation of sustainable agricultural practices (Figure 1). 

The use of nanotechnology in the creation of nano-pesticides is another key application that is helping horticulture crops to thrive. Thus, the use of nanotechnology in agriculture methods will undoubtedly set the standard for achieving the long-term goal of agro-technologically producing sustainable crops. 

## 2. Agriculture in the 21st Century

Inhumane levels of hunger currently affect over 800 million people, and by 2050, the global population is projected to be close to 9.7 billion [8]. A rapid increase in food poverty has the potential to spark conflict, ecological instability, and economic unpredictability. Reduced agricultural productivity, nutritional scarcity, and climate change are some of the biggest challenges agricultural scientists face today. Consequently, it is believed that current agricultural output need to be increased by as much as 50–70% to fulfill both present and future food requirements [9]. Heavy fertilization of agricultural soils, tillage methods, fossil fuel usage, and livestock manure are the principal sources of greenhouse gas emissions, and hence represent a major contributor to climate change. Soil fertility, food security, and the environment have all declined as a result of extensive crop production coupled with an overreliance on chemical fertilizers and pesticides. Increases in temperature and atmospheric CO_2_ levels, as well as changes in rainfall patterns, pose a danger to agricultural productivity and lead to drastically reduced crop yields [10]. As a result, individuals experience a wide range of health issues due to the instability and poor quality of the food they consume. Unpredictable seasonal and geographical changes interrupt plant life cycles, which means that climate change affects crop nutritional quality. One of the greatest issues facing contemporary agriculture is meeting the world’s present and projected food needs. Defending natural resources to support increased agriculture while reducing adverse environmental consequences is a huge challenge that will require a concerted effort from interdisciplinary researchers. 

Crops that are subjected to biotic and abiotic stressors experience a broad variety of diseases, many of which negatively affect crop output and nutritional quality. Over the past sixty years, artificial chemical fertilizers have been at the forefront of efforts to revive agricultural output and meet surging global food demand. Scientific and agricultural studies show that using agrochemicals like phosphate, ammonia, nitrate, or urea compounds in farming has disastrous effects on ecological health when used extensively and over extended periods. This has become a significant contributor to issues such as soil and water contamination, eroded landscapes, hunger, low fertility, inefficient water retention, and the disturbance of native soil biodiversity [10]. Unfortunately, these agrochemicals do not stay out of the hydrosphere and contribute to eutrophication. Even more problematic is the fact that water constraint compounds the impact of nutrient pollution on soil quality, in addition to increasing emissions of greenhouse gases, eutrophication in aquatic environments, and soil salinization, all of which pose serious threats to food safety and quality. The latest research [11] has shown that a sizeable fraction of synthetic fertilizers are not consumed by plants but instead escape into the environment or drainage sources like waterways. Plant-growth-promoting rhizobacteria (PGPR) as biofertilizers have been advocated as being among the most sustainable and resilient methods to boost crop production. By providing essential nutrients, they stimulate plant growth via several direct and indirect pathways.

Nitrogen fixation, exopolysaccharide synthesis, the production of siderophores and phytohormones along with nutrient solubilization are all examples of direct mechanisms. Through colonization of the rhizosphere, they provide plants with stress tolerance and resilience [12]. The development of sustainable agricultural techniques should be prioritized, since we cannot entirely remove the use of agrochemicals; nevertheless, we may pick other ways to make crop production more efficient. Designing, characterizing, and manufacturing substances by controlling their sizes and shapes at the nanoscale constitute what the Royal Society calls “nanotechnology” [13]. 

Nanotechnology is a relatively recent scientific idea that makes use of methods and materials that change the physio-chemical properties of a substance. The integration of these two fields has led to revolutionary developments in the agricultural sector [13]. Due to the nature of climate change, nanotechnology has a wide range of possible uses and benefits. This new paradigm has matured to the point that it can be used in real-world settings. Soil structure, insects, infections, pollutants, and the distribution of pesticides, fertilizers, nutrients, and genetic components will be managed, all to exert a significant impact on agriculture [14]. Nanoparticles (NPs) operate as triggers, kicking off several defensive mechanisms in plants subjected to stress. Many different fields of application have welcomed this fast-developing technology, including plant regulatory surveillance, improved fertilizer usage efficiency, accelerated plant development, and regular pesticide release [15]. Surprisingly, a nanoparticle-derived strategy has gained favor and effectiveness in agriculture for crop sustainability, beating out bio-pesticides and other fertilizers due to the particular features of nanoparticles, such as their large surface area, high solubility, and light weight [16]. 

**Figure 2:**
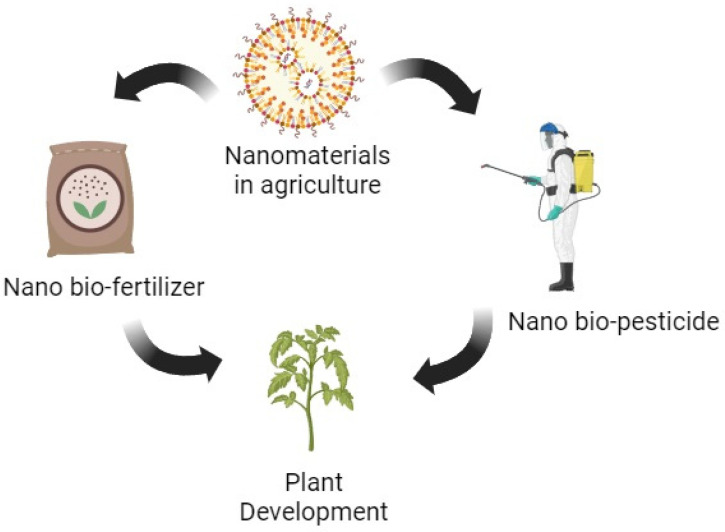
Application of nanomaterials in plant development.

Nanotechnology, biotechnology, and other scientific fields are being integrated into agriculture to transform traditional farming practices into ecologically responsible and economically viable “precision agriculture” that can feed the country’s expanding population. The term “nano-biofertilizer” (NBF) refers to a compound made up of both nanoparticles and biofertilizers. Nanosized biofertilizers (1–100 nm) are encapsulated in a suitable nanomaterial covering, and then the resulting product is manufactured [17]. They relieve the effects of environmental stressors by controlling the distribution of nutrients, moving them to their intended destination. Their most notable qualities are a lower need for synthetic fertilizers, higher yields with less time and effort, lower environmental impacts, lower costs, and more nutrient availability and absorption [18]. It was shown that NPs can affect plant–microbe interactions in two ways: directly, by altering the accessibility to nutrients in the rhizospheric soil, and indirectly, by stimulating microorganisms. Nano-biofertilizers improve the soil microbiota, increase N_2_ fixation, improve the solubilization of phosphate, and increase the synthesis of plant-growth-regulating hormones (Figure 2). 

Nano-biofertilizers, whether applied on the leaves, seeds, or soil, have a unique ability to enter plants [11]. When used as a coating or immobilization substrate for biofertilizers, the nanomaterial has the potential to enhance the dissolving and diffusion of insoluble nutrients in the soil, raise nutritional bio-availability to soil and plants, and allow for the slow and regulated discharge of nutrients that are directly internalized and absorbed by plants. For a plant’s development cycle, the available nutrients are gradually increased with regulated release. When farmers have accurate data on the weather, soil, and other factors, they can run their farms more effectively [17]. As one example, farmers may use nano-devices to monitor crop health and catch issues early on before they become noticeable. Devices of this type could be able to respond quickly to a wide variety of situations by implementing suitable control methods [19].

Since they are so small, NBFs can effortlessly pass through plant tissue and rapidly reach their goal despite gravity’s best efforts to slow them down. When applied to crops, nano-biofertilizers combine the benefits of bio-inoculants with nanoparticles to increase yield [20]. Microbe-based nano-biofertilizers have yet to be extensively approved and marketed due to a lack of information regarding the interactions between biofertilizers, nanoparticles, and plants. Synthetic nanofertilizers have been the subject of recent studies in both the physical and chemical sciences. Therefore, research into the development of more accessible nano-biofertilizers is needed so that they may be marketed to farmers. The purpose of this review is thus to shed light on the interaction of nano-biofertilizers with the plant system. It is important to note that the negative impacts of physio-chemically manufactured NBFs have been extensively addressed, and green production approaches using a wide range of microorganisms and microbial compounds have been developed as a result [21]. We need to assess the results of climate change on plants, soil, and the environment. We will address the function of NBFs and the principal mechanisms by which they affect plants, as well as their interaction with rhizo-microbiomes that promote plant development and soil fertility. Third, many inorganic and organic NBFs and nano-aided developments are characterized, and their roles are discussed in the context of precision farming for pulse, cereal, and millet crops.

## 3. Data Collection and Analyses

**Figure 3:**
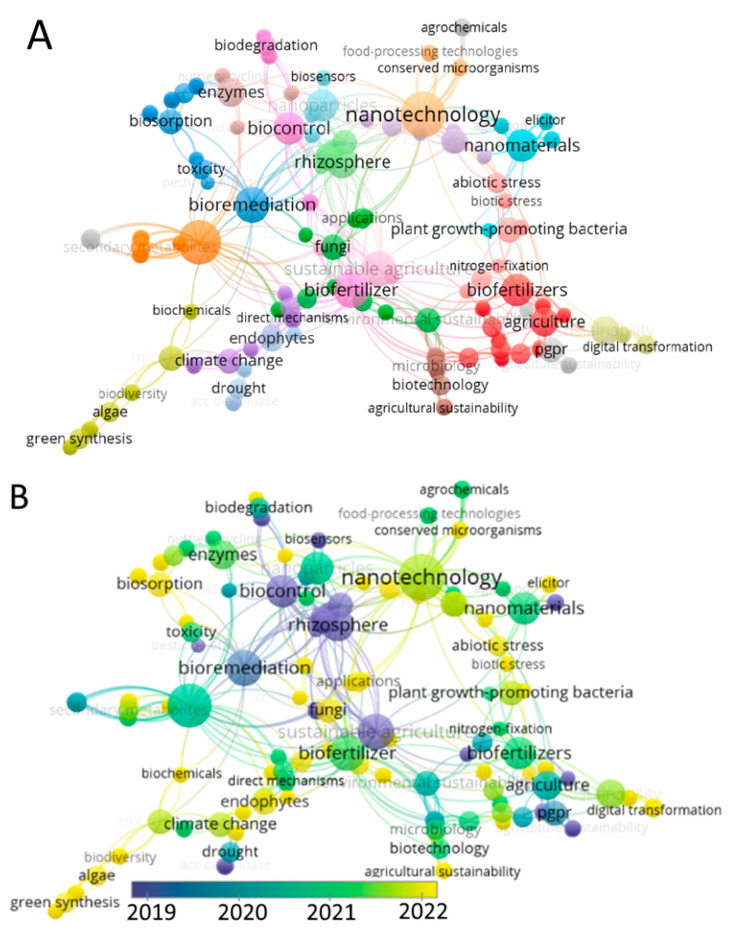

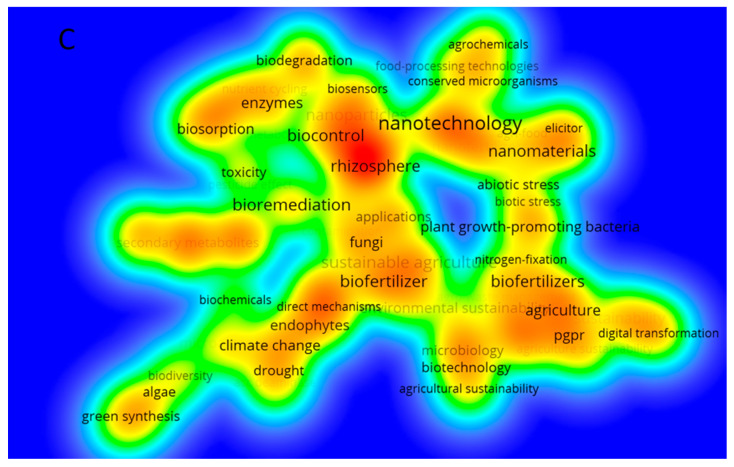
Graphs of high-frequency keywords over time and keyword citation network clustering used in bibliometric analysis. (**A**) Network visualization, (**B**) overlay visualization, and (**C**) density visualization. Bibliometric network term lines, different colors, and circles refer to the strength of their connectivity. The higher the weight of an item, the larger the label and the circle of the item. As the weight of an item increases/decreases, both the label and the corresponding circle representing the item also increase/decrease in size. A timeline is represented by a gradient from red to blue, with red denoting older publications and blue denoting newer ones. The gradient serves as a visual indicator of time passing. Red denotes significant or noteworthy elements, such as notable citations, seminal publications, or sources that are frequently mentioned. While the newer publications, rising trends, or sources that are regarded as fundamental in a certain field may all be represented by the colour blue. Additionally, it might indicate sources having a particular subject or substance.

In the present study, we looked for “nano biofertilizers”, “plants”, and “agriculture” in the Web of Science and found 191 relevant publications. The search was performed using VOS viewer 1.6.18. An abundance of publications is a strong indicator that may be used to guide the selection of a research topic. The number of publications about NBFs and citations relating to them increased quickly beginning in 2020, reaching a peak in 2022. The keyword list may be broken down into individual fields of study. A network visualization map showing the co-citation of the most-cited terms from 191 publications published from 2019 to 2022 is shown in Figure 3. The majority of current research is focused on the creation of new NBFs, and the portrayal revealed that agriculture, nanotechnology, and plants are among the most investigated fields in the framework of NBF studies. 

## 4. Nanomaterial Synthesis

The synthesis of nanomaterials is extremely sensitive to substrate temperature, reactor pressure, nanomaterials, and gas-phase chemical composition. Constant effort has been put into discovering new ways to create nanoparticles with enhanced properties, including form, size, commercial feasibility, and usefulness. Two main types of procedures may be used in the synthesis of nanomaterials, each of which may use biological, physical, chemical, or hybrid processes [22]. 

### 4.1. Top-Down Approach

This process entails the constant breaking down of large pieces of material into smaller ones. This strategy incorporates several decomposition processes, including milling, physical vapor deposition, chemical vapor deposition, and others. Nanomaterials including graphenes, fullerenes, and carbon tubes are created by applying this process. Chemical vapor deposition (CVD) is commonly employed for the production of carbon-based nanomaterials [23]. This is a complicated process that calls for a chain reaction to occur between the gas and the surface. Among the many uses for the CVD process are the creation of composite materials, the production of powders, the deposition of thin coatings on surfaces, and the synthesis of pure, bulky substances. 

Limitations of the top-down technique include milling, CVD, and physical vapor deposition—complex and sometimes sophisticated procedures that gradually reduce bigger materials into smaller ones. For the manufacturing of carbon-based nanomaterials, CVD especially calls for complex gas–surface reactions [24]. While this process is useful for creating thin coatings and composite materials, it may have difficulty precisely controlling the structures and characteristics of the materials.

### 4.2. Bottom-Up Approach

Bottom-up synthesis, also known as biological synthesis, green synthesis, and spin coating, is the opposite of top-down synthesis, since it uses more complicated molecules to make nanoparticles. The effectiveness of this procedure is dependent on the incorporation of material components that have effective self-assembling qualities when it comes to the development of nanostructures. This technique is used on a regular basis in the generation of quantum dots during epitaxial growth as well as in the colloidal suspension of nanoparticles [25]. The success of the bottom-up strategy depends on the accessibility of material elements with built-in self-assembling properties for nanostructure formation [24]. 

### 4.3. Nanoparticle Fabrication

Creating a nano-biofertilizer involves three major steps: (1) cultivating the biofertilizer culture; (2) encasing the culture in nanoparticles; and (3) evaluating the product’s efficacy, quality, and purity, as well as its reliable storage [26]. The bulk material is broken down into smaller particles to form nanoparticles. By utilizing a size-reduction process, large quantities of material may be broken down into minute particles known as nanoparticles [27]. Importantly, unlike the easy transformation of bulk materials into nanoparticles, shrinking living beings is not feasible. The shrinkage of living beings to the nanoscale range is lethal. Bacterial, fungal, and plant cells have a typical size in the range of micrometers [28]. The production of nanomaterials employs these live cells as a means of scaling down the size of the starting material to the nanometer range. Synthesized NPs from silver and other metals are efficient antimicrobial agents that can be fabricated with the help of microorganisms, namely *Bacillus megaterium*. Another advised approach is NP aggregation, since these particles may safely enter the cell wall of plant-growth-promoting microbes. The attachment of NPs has the potential to alter the cell’s size and shape, which might accelerate the maturation of the microbial cell [29]. The bacterial cell-free supernatant is gaining popularity as a well-known agent for the green synthesis of nanoparticles. Eliminating steps like sonicating cells to break down their cell walls, centrifuging to separate biomass, and washing methods to purify NPs makes this method very scalable [30]. 

Mycological NPs were produced by cultivating fungi in MGYP broth comprising maltose, glucose, yeast, and potato, and recovering the fungal colonies via sifting. The collected biomass was treated with AgNO_3_ and then incubated in the dark to create Ag-nanoparticles. After being confirmed using a UV-Vis spectrophotometer (350–650 nm), the manufactured silver NPs were freeze-dried and stored for further use [16]. The nano-biofertilizer was created by combining metals with bioorganic components like auxin. The process involves isolating auxin from bacterial cells. A 0.22 m membrane was used to filter the cell supernatant before it was diluted to 200 g/mL with ultra-pure water. When synthesizing monometallic nano-compounds, a 2 mM (of metal) aqueous solution is combined with 50 g/mL auxin, while when preparing bimetallic nano-compounds, 1 mM (of metal) is added to two 20 mM (of metal) solutions. The pH 4.8 reaction mixture is then heated in the dark at 45 degrees Celsius in a water bath for 5 h (no agitation) [10]. The endophytic bacteria *Paenibacillus polymyxa* DEBB B-358 with auxin complex was used in a supernatant solution for the efficient development of mono- and bimetallic iron and manganese NPs [31]. 

### 4.4. The Role of External Factors in Nanoparticle Synthesis

Many physiological factors, including the type of microorganism used in the reaction, the reaction temperature, the pH, the pressure, the incubation time, the concentration of metal salts, and many others, control NP biosynthesis [32]. The following is a summary of several key factors that influence NP biogenesis. The morphological features of synthesized NPs are significantly affected by the pH of the synthesis medium.

1. Temperature: The temperature of the synthesis medium is the determining factor in the type of NP that is produced. Unlike the green method, which operates at temperatures as low as 100 degrees Celsius, the physicochemical method necessitates temperatures of 350 degrees Celsius or higher for NP synthesis, rendering it incompatible with microorganisms [33].
2. Pressure: The size and form of synthesized NPs are controlled by pressure. At atmospheric pressure, biological agents are much more efficient at reducing metal ions [34].
3. Incubation period: The length of time that the reaction medium containing biological entities is allowed to incubate for determines the kind and quality of NPs produced. Changes in particle size, number, and size distribution as a result of storage, shelf life, and other factors are all governed by the passage of time [35].
4. Expenditure: The most significant component that has to be improved is the economic costs connected with NP synthesis to raise their market value. The physical and chemical processes used to achieve the high yield were quite expensive. However, large-scale green synthesis is feasible within an optimal cost range for farmers [36].
5. Proximity: The electrical charge on particles, substrate–NP interactions, and the magnetism of NPs are all governed by the proximity effect. Isolated or singular materials display dynamic changes in behavior when subjected to social interaction, which could be exploited for more nuanced NP design [37].

Before NPs can be used in any way, they must be cleaned up after they are manufactured. The separation and enrichment of the produced NPs, together with the removal of non-reactive bioactive compounds, are typically accomplished via repeated washing and high-speed centrifugation. After the cell wall has been broken down, additional purification methods such as ultrasonication or contact with suitable detergents are required to release NBFs created within the cells. The raw material concentration and size, as well as process factors like light, storage conditions, etc., all have a role in NBF synthesis and stability. The number and kind of extracellular and intracellular enzymes released by bacteria also have a significant role in NP production. 

### 4.5. Types of Nanomaterial

Nanomaterials and nanoparticles designed for agriculture must contain a specific mineral that the plant can use to grow and produce greater yield. Nanotechnology’s advantages include the ability to produce selective substances in nanosized dimensions (100 nm), which allow them to permeate deeply into plant tissues or soil particles, where they can contribute essential nutrients [38]. Micronutrients like manganese (Mn), boron (B), copper (Cu), silicon (Si), zinc (Zn), nickel (Ni), iron (Fe), and molybdenum (Mo), and macronutrients like N, P, K, magnesium (Mg), calcium (Ca), and sulfur (S) can all be found in nanomaterials, as mentioned in several studies [39]. 

Nanofertilizers have also been developed using organic nanomaterials such as lignin, lipids, urea, starch, chitosan, cellulose, zeolites, polymers, and others. Their worth for agricultural production is enhanced by the fact that these substances not only serve as a direct amendment of deficient nutrients, but also exhibit anti-pathogenic action. If we want to avert any potential toxicity or secondary repercussions, we must investigate their long-term relationship with the crops and the environment [40]. 

#### 4.5.1. Carbon Nanotubes

Carbon is a fascinating element, since it can take on a variety of forms with distinctly different characteristics. Diamond and graphite are two of the most well-known allotropic forms of carbon. They are employed in many different kinds of scientific and technological items, and could not be more different from one another [41]. Several new low-dimensional carbon forms have been discovered, expanding the catalog of carbon allotropes. Nanoparticles made from naturally occurring carbon are very small. The vast majority of them have been intentionally created. Graphene, carbon tubes, and other allotropes have more practical uses. Traditional taxonomies group them by the geometry of their structures. Carbon tubes, nanotubes, and fullerenes are complete spheres or ellipsoids. Despite the proven usefulness of carbon nanoparticles in crop development, improving the efficacy of composite materials, improving plant health, environmental cleanup, fuel cell supercapacitors, and electro-optic sensors, their usage is limited in a variety of systems, including agriculture [42]. The stability of metals linked to carbon nanoparticles in the system is not well characterized, and there is a lack of evidence on the effect on nutrition or changes to genetic material. With growing exposure to the environment, it is crucial to highlight the consequences of these substances. Common examples of carbon-based nanoparticles include fullerenes and carbon nanotubes (CNTs) [43]. Their structure and features, including durability, strong electron affinity, conductivity, and adaptability, provide them with a broad variety of potential uses. They are both capable of translocating inside a plant, meaning they may be able to travel from one section of the plant to another. Since they help plants to take in more of the nutrients they need, they are widely utilized in agriculture to boost crop yields.

#### 4.5.2. Hybrid Nanocomposites

Organic or inorganic conjugates with unique features are of great interest for a wide range of applications in biofertilizer production for crops. Because of the incorporation of additional nanomaterials, they are more effective and versatile than single-type nanomaterials. Hybrid nanomaterials are created when two or more nanoparticle types are combined, such as carbon-based, metal, or polymeric nanoparticles [44]. Consequently, as a result of their improved biocompatibility, they are increasingly employed in biological applications such as agriculture, medicine, and medication delivery [45]. 

#### 4.5.3. Metal-Based Nanoparticles

Metal-based nanoparticles are crucial in a number of scientific and technological applications. Bottom-up and top-down synthesis are the most popular methods for creating these nanomaterials; however, other physical, chemical, biological, and hybrid processes are also possible. Fe, Mn, Mg, Ni, Zn, Cu, Ag, and Co are some of the most often synthesized metal nanomaterials [46]. Several methods, such as thermal breakdown, microemulsions, and co-precipitation, are used to create these nanoparticles. Magnetic NPs are superior to other nanoparticles because of their unique physicochemical qualities; for example, they are entirely fabricated from precursors that provide them with localized surface plasmon resonance (LSPR) features [47]. These nanoparticles may be changed with a wide variety of functional groups, opening up the possibility for their usage in diagnostics, gene transfer, non-invasive imaging, agriculture, and biotechnology [48]. They could open up new possibilities for the binders of agrochemicals, insecticides, and fertilizers. They help seeds to germinate, promote plant development, safeguard crops, and boost overall harvest success. Although nanotechnology and biotechnology have opened up boundless possibilities for agriculture and crop management, the influence of nanoparticles is highly dependent on their size, composition, concentration, surface charge, and the sensitivity of plant species to them. Metals and metal oxides at the nanoscale have been integrated into a wide range of agrochemicals to perform a wide range of tasks. Avermectin was preserved in silicon dioxide (SiO_2_) nanospheres, but its degradation was accelerated by applying nanosized titanium dioxide (nTiO_2_) to the nanosphere surfaces [10]. The role of nTiO_2_ and zinc oxide (ZnO) in a recently patented grass fertilizer was described as “radiation managers,” but their precise function remains unknown. Bactericidal efficacy against *Liberibacter crescens* and *L. asiaticus* communities has been established in a ZnO-based nanoformulation [49]. 

Nanoformulations often include these metallic nanoparticles. In nanoformulations, silver nanoparticles are exploited for their toxicity against infections. Surface coatings containing a combination of silver nanoparticles and amphiphilic hyperbranched macromolecules have been demonstrated to be efficient against a wide variety of diseases [50]. Significant antifungal action was demonstrated using silver nanoparticles and fluconazole (a triazole fungicide) against *Phoma glomerate*, *Trichoderma* sp., and *Candida albicans* [51]. *Sclerotium rolfsii* in mung bean was significantly stunted by silver nanoemulsions, and this nanoemulsion was also shown to have a potent inhibitory effect on *Bipolaris sorokiniana* and *Magnaporthe grisea* [52]. 

Nanoemulsions of titanium dioxide were the focus of research by [53], who sought to develop a nanofungicide for use against plant pathogens.

#### 4.5.4. Nanoscale Polymers

Polymer-based nanoparticles include nanospheres, nanocapsules, and other substances. More and more often, they are being put to use in fields as diverse as biology, medication delivery, agriculture, diagnostics, and imaging [54]. Insecticidal formulations based on NPs include chosen active chemicals optimized for use against insects and often encapsulated within NPs for transport and increased efficacy. The chemical ingredients can be encased in a capsule formed from sodium alginate, silica, polyethylene glycol (PEG), or chitosan or in an emulsion, suspension, polymer sheet, or gel [55]. Nanocapsules are vesicular or reservoir-like structures made up of a hollow interior lined with a polymer covering or membrane. Tebuconazole- and carbendazim-loaded polymeric and solid lipid nanocapsules have been employed as nano fungicides [56]. Methyl methacrylate (MMA) and styrene (St) copolymer nanocapsules containing lansiumamide have also been investigated, and it has been shown that LB-loaded poly (MMA-co-St) nanocapsules (NCLB) have nematicidal action. Insecticide nanoformulations necessitate a nanocarrier system that allows for the timed release of the core active chemical, so that an effective concentration of the insecticide may be maintained throughout the development of the insect. 

#### 4.5.5. Nanoemulsions

Emulsions with droplet sizes between 20 and 200 nm (also known as mini-emulsions, sub-micron emulsions, and ultrafine emulsions) have dimensions that overlap with microemulsions. In comparison to microemulsions (20%), nanoemulsions have a lower surfactant concentration (5–10%) and are hence metastable. When applied to crops, nanoemulsions increase the bioavailability of agrochemicals by increasing their solubility. Nanoemulsion pesticides may have advantages over traditional methods due to their high surface adhesion, deep penetration, and wide scope of use [57]. 

Pesticides and herbicides have been used in the creation of several nanoemulsions. Nanopesticides in the form of nanoemulsions may be an efficient means of delivering water-insoluble pesticide active components [58]. 

Nanoemulsions also have uses in the agri-food industry for the processing and storage of collected goods. For the insecticide beta-cypermethrin (β-CP), a similar green oil-in-water nanoemulsion was created. Methyl laurate was employed as the oil phase, while alkyl polyglycosides (APGs) and polyoxyethylene 3-lauryl ether were used as mixed surfactants. Active components of agrochemicals can be encapsulated in nanoemulsion droplets to provide a formulation with little loss of efficacy [59]. As a second use, nanoemulsions are created to transport naturally occurring chemicals with insecticidal characteristics that are either not water-soluble or very slightly water-soluble [60]. The potential of nanoemulsions to combat a wide range of plant diseases has already been emphasized by researchers [61]. Weeds and grasses compete with crops for sunlight, water, and nutrients, and hence certain nanoemulsions were utilized to kill them [62]. A nicotine carboxylate nanoemulsion created from a sequence of fatty acids (C10C18) and a surfactant [63] had a mean particle size of 100 nm, and it had a unimodal size distribution. Lethal time 50 was used to determine the bioactivity of the pesticide formulations against adult *Drosophila melanogaster* (LT50). 

#### 4.5.6. Nano Dispersions

Nanocrystals in a liquid medium are called nanodispersions. Nanodispersions can improve the solubility of active substances that are not very water-soluble [64].

#### 4.5.7. Nanoencapsulation

In nanotechnology, encapsulation is a common practice. The properties of nanoencapsulation are useful in a wide range of agricultural applications, from the control of insect pests with encapsulated nanoinsecticides, to the promotion of the correct assimilation of chemicals into plants, to the protection of host plants using nano-encapsulated DNA or chemicals. Nanopesticides often employ biodegradable polymers like polysaccharides and proteins for their nanoencapsulation [65]. Another potential field is the use of nanoencapsulation in nano fungicide formulations. Experiments have demonstrated that encapsulating biocides like avermectin and 2,4-D in organic or mineral nanospheres or adsorbed onto the surfaces of nanoclays or nanoporous materials like chitosan or zeolites may significantly slow the release rate of these chemicals [66]. Improved solubility, selectivity, permeability, and stability are only some of the benefits that may be gained by encapsulating insecticides in nanoparticles. Built-in switches and timers control the release and accessibility of pesticides. It was discovered that the phytotoxicity of herbicides on crops might be decreased with the use of nano-encapsulated herbicides for the suppression of parasitic weeds [67]. 

#### 4.5.8. Liposomes

Using chitosan as a coating on the basic liposomes of etofenprox or alpha-cypermethrin, the authors of [41] were able to produce a nanocarrier system that lasted for an extended period of time. By increasing the concentration of the coating material and the intrinsic surface charge of the nanoliposomes, it is possible to create a long-term, continuous release of etofenprox and alphacypermethrin that are entrapped in chitosan-coated nanoliposomes. It has been shown that formulations that are made up of liposomes are effective in killing insects [68]. Bulk pyrifluquinazon was most hazardous at 50 and 25 ppm, respectively, two days after treatment. However, a controlled-release nanoformulation of the drug that was loaded with chitosan was more effective fourteen days after treatment. It was shown that the uncoated fungal metabolite and the fungal spores, as well as the entomopathogenic fungus *Nomuraea rileyi* metabolite encapsulated in a chitosan nanocarrier, had greater pesticidal activity against the pest *Spodoptera litura* [69]. 

#### 4.5.9. Clay Encapsulations 

Because of their low cost, longer release, and increased interaction with plants, clay nanotubes, also known as halloysite, have been created as carriers of pesticides. This has the additional advantage of minimizing the effect that pesticides have on water streams [70]. Nanomaterials such as anionic inorganic or organic layered double hydroxides (LDH), natural inorganic clays, organo-modified clays, which can be called hybrids or composites, and other materials are examples of nanomaterials whose mechanistic interaction with pesticides is well documented even at the molecular level [71]. 

#### 4.5.10. Encapsulations

Because they make it easier for chemicals to enter the bodies of insects, biological control efforts often make use of nanoformulations that are generated from microorganisms such as bacteria and fungi. The nanoformulated pirimiphos insecticide has a long shelf life, since it can be stored in the dark without losing any of its effectiveness [72]. When plant extracts were made into nanoparticles, their effectiveness as pesticides rose by more than a factor of five against insects that were resistant to conventional pesticides [31]. Nanoformulated permethrin, which is produced via the evaporation of an oil solvent in water, has the potential to significantly enhance Culex larval management [73]. Utilizing chitosan polymer nanoparticles allowed for the examination of the pathogenicity of *Bacillus thuringiensis* in relation to Anopheles larvae. It was found that soil that had been planted with cyanobacteria as nanocarriers for the stimuli-controlled administration of avermectin displayed improved performance as well as a greater photostability against UV radiation. In order to increase plant production and output, several plant pathogenic bacteria, such as *Agrobacterium tumefaciens*, *Agrobacterium rhizogenes, Rhodococcus fascians, Pseudomonas syringae*, and *Streptomyces* sp., have been used. 

#### 4.5.11. Nanospheres

Nanospheres are a homogeneous system as opposed to nanocapsules because the bioactive component is evenly distributed throughout the polymer matrix [74,75]. According to [76], nanospheres may act as controlled-release carriers and protective reservoirs, extending protection over a broader region and lowering leaching losses. Nanospheres that contain the pesticide azadirachtin are strong enough to withstand exposure to UV light without degrading [77].

#### 4.5.12. Nanomicelles

Because they can form a core–shell shape in water, micelles are amphiphilic block copolymer colloidal particles that are widely utilized in the delivery of water-insoluble agrochemicals [76]. Micelles are created when amphiphilic polymers clump together. They enclose the active ingredient and shield it from microbial and environmental deterioration, enabling optimal release over a longer period of time than the commercial formulation. In order to distribute the pesticide mancozeb to certain insects, the use of micelles was investigated in [76]. Tomato early blight has been prevented by utilizing mancozeb nanoformulations made with amphiphilic polymers [78]. Ref. [79] grafted the amphiphilic ricinoleic acid to the critical micelle concentration CMCS to produce a lipophilic azadirachtin micelle solution in water. Effective pesticide combination control utilizing micelles and hydrogels has been shown. 

#### 4.5.13. Nanogels

A newly identified class of NMs with intriguing potential are nanogels. Nanogels have a number of properties with nanoparticles; however, they are substantially smaller than hydrogels [80]. When a hydrogel and crosslinked hydrophilic polymers are mixed, nanogels are created [81]. The majority of nanogels are smaller than 100 nm; however, by varying the solvent quality and volume percentage, their dimensionality may be changed in a number of ways 2019 [82]. There are numerous methods for creating nanogels, including the physical assembly of interacting polymers and template-assisted nanofabrication. The combined effects of copper and pheromone against *Fusarium graminearum* and fruit pests increased the antifungal efficacy of chitosan nanogels [83]. They differ according to their physio-chemical characteristics, stimulus reactivity (temperature, pH, etc.), biomolecule encapsulation potential, and colloidal stability [84]. Chitosan, poly(ethylene oxide), poly(vinyl alcohol), poly(ethyleneimine), poly(vinyl pyrrolidone), alginate, and poly(vinyl pyrrolidone) are examples of common NGs. Hybrid structures made from polymeric or non-polymeric materials may be used to create NGs. Interpenetrated networks (IPNs), core–shell particles, and copolymers are examples of polymer nanogel composites that include nanoscale elements like magnetic NPs or carbonaceous NPs [85]. NGs with a core–shell are often a good bet. Due to the following restrictions, it is feasible to draw the conclusion that the potential and benefits of NMs are being misused in plant research disciplines: despite the urgent need for safe, secure NM synthesis and design to minimize the interference with plant growth and development, we know very little about the specific processes by which plants mobilize and absorb NMs [86]. 

#### 4.5.14. Dendrimers

Natural plant fibers, particularly in fiber-reinforced polymer matrix composites, have long helped agriculture. These composites are widely used; however, their poor mechanical qualities make applications difficult. Dendrimers—hyperbranched polymers—solve this problem [87]. Their low viscosity and great reactivity set them apart. The multireactive end groups strengthen plant fibers. Modifying these fibers improves fiber–resin matrix interface bonding. Synergy improves composite mechanical characteristics and performance. Dendrimers are crucial to addressing composite mechanical characteristics. Dendrimer-based modifications may improve the efficiency and durability of polymer matrix composites in many applications by improving surface interactions and maximizing natural plant fibers.

## 5. Properties of Nanoparticles

**Figure 4:**
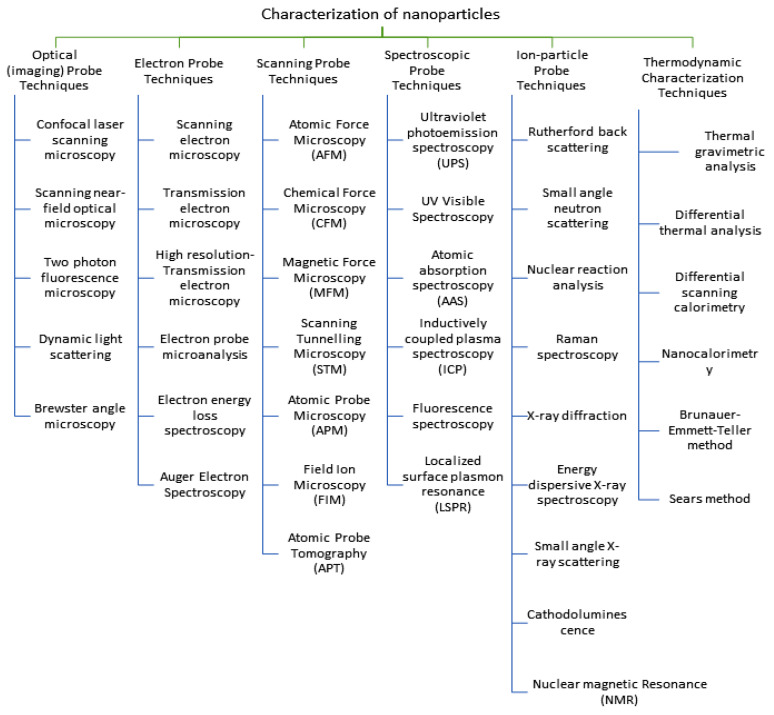
Schematic representation of various techniques used for the characterization of nanoparticles.

In Greek, the prefix “nano” means “dwarf.” As a noun, “nano” connotes diminutive dimensions. Nanotechnology involves the study, research, and use of structures and materials with dimensions on the nanometer scale (100 nm) or less [88]. According to ASTM standards, the diameter of a nanomaterial (NM) is anywhere from 1 to 100 nm [89]. The nanoscale nature of these particles is not the only thing that sets them apart from conventional materials, though; their increased surface area, controlled pore size, heightened reactivity, and shape are also notable. Researchers have studied the use of nanoparticles for agriculture development [90]. It has been shown that NMs have antibacterial, insecticidal, fertilizing, and herbicidal effects, among others. Nanoparticles can be characterized as having various properties using different techniques listed in Figure 4. 

## 6. Influence of Nanoparticles on Plant Life

The realization that significantly improved seed germination and development, as well as a plant’s capacity to withstand biotic and abiotic challenges, require a significantly more efficient system, presumably decreased in size, has spurred recent interest in the use of nanotechnology in plant research [85]. For example, silver [90] and gold (Au) nanoparticles have been used for a variety of beneficial applications in the study of plants. Despite its simplicity, the chemical manufacturing of NPs involves the use of hazardous chemicals and expensive expenses. However, more environmentally friendly methods, such those that use microorganisms and plant extracts, have been created. Because of the exceptional light-absorbing, catalytic, and electricity-conducting capabilities they display, studies of oxidized NMs including MgO, ZnO, TiO_2_, and CaO NMs have been intensively performed [91,92,93,94]. Due to their ability to react to environmental cues, their biocompatibility, and their ease of production, polymeric NMs are more often utilized now than when they were initially created [95]. There are additional core/shell NPs available, chosen for their unique features. A broad range of components, including inorganic/inorganic, organic/organic, and inorganic/organic combinations, may be used in their synthesis. 

## 7. Plant–Nanomaterial Interactions

**Figure 5:**
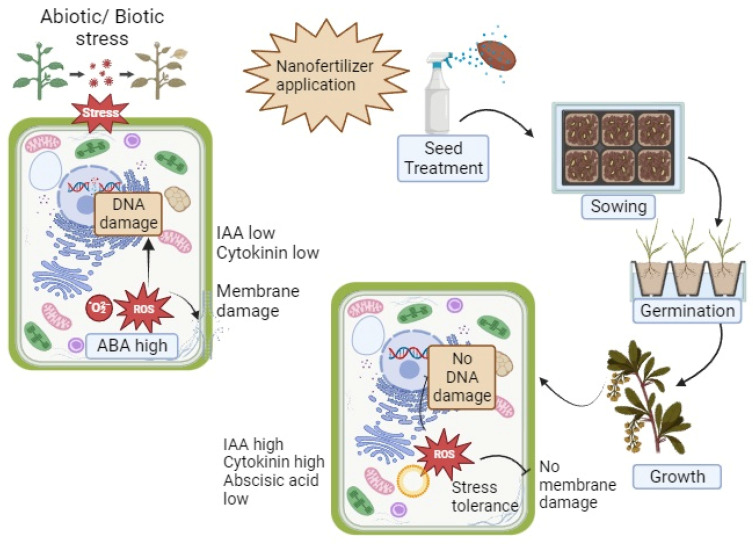
Mechanism of action of nano-biofertilizers on plants under stress conditions (constructed by authors using BioRender, Toronto, ONT, Canada).

Plants have had to deal with environmental NMs, both metallic and organic, throughout their evolutionary history [96]. But as engineered nanomaterials (ENMs) become more widely used and produced, the number of plants that are exposed to NMs through both accidental and intentional means is expected to increase dramatically. This sort of elevated exposure might be harmful to the environment and, by extension, to human health [97]. Humans, microorganisms, mammalian cell lines, and other indicator species have all been used in numerous investigations of the toxicity of NMs. However, the impact and toxicity of NMs on plants have received far less focus. It is well established that plant cells take up NPs and then spread them throughout the plant. Ecotoxicologists agree that this might be a route via which NPs enter the food chain and accumulate. Research on NM interactions with plants is crucial because of the role NMs play in the terrestrial food web [98]. For this reason, understanding how plant cells take up NPs in their bodies is crucial [99]. The response of plants to NP exposure has been demonstrated to be nuanced and complex, influenced by several parameters including plant size, NP concentration, duration of exposure, and the type of NP [100]. The consequences of plant–NP interactions, however, are contingent on a wide range of other circumstances (Figure 5). Additional factors to think about include plant physiology and the method through which NPs are taken up by plants [101]. 

Although ENMs may be applied to the roots or any other vegetative portion of the plant, they are most often seen in the leaves when given to plants. The absorption of passively absorbable NPs at the shooting surface is made possible by the micro- or nanoscale exclusion sizes of the natural plant apertures, such as the hydathodes, stomata, bark texture, and stigma. More knowledge of plant physiology and anatomy is required to completely understand the dynamics of plant–NP interactions [102]. On the surface of a plant’s shoots, a cuticle consisting of biopolymers and associated waxes creates a lipophilic barrier and restricts access to natural apertures. Although it has been shown that nano TiO_2_ may lead to cuticle holes [103], it has been discovered that this barrier is presently a very impermeable layer to NPs. By keeping the NPs there for a longer time and increasing their contact with plant tissues, the trichomes of a plant may change the dynamics of NP interaction at the plant’s surface. The internalization of NPs is facilitated by wounds or damage in the hypogeal and aerial sections of plants [27]. It seems that elements other than the NPs themselves have an impact on how well plants absorb NPs following delivery. One kind of microorganism that interacts symbiotically with plants and affects their capacity to absorb nitrogen is mycorrhizal fungi. 

## 8. Intracellular Nanomaterial Mobilization

NMs may activate the plant’s defenses in one of two ways after they have gotten beyond the outer defense system, whether by means of hypogeal exposure or aerial exposure. Mobilized cells have the option of becoming apoplastic or symplastic. Symplasts, as opposed to apoplasts, employ plasmodesmata, which are specialized structures, and sieve plate pores to move solutes and water across the cytoplasm of adjacent cells [104]. Apoplastic transport travels outside of the plasma membrane, passing through the xylem, intercellular spaces, and neighboring cells’ cell walls. According to research [105], apoplastic transport encourages NMs to travel radially, transports them to vascular tissues and the central cylinder of the root, and then drives them upward in the plant’s aerial section. However, since NMs migrate through the symplastic route after exiting the xylem and before accessing endodermal cells, the Casparian strip has a higher concentration of NMs. This holds true for the regulated distribution of NMs as well [106]. The cells in a particular area must be repeatedly penetrated by NMs in order to carry out symplastic transport. Therefore, since plant cells have a thicker cell wall than animal cells do, which physically prevents NMs from entering the cell, the intracellular transport of NMs in plants is considerably more challenging than in animal cells. A porous, selectively permeable environment with a typical width of less than 10 nm and a maximum diameter of 20 nm is provided by the microfibrils, scaffold proteins, and hemicellulose/cellulose that make up the cell wall [107]. Many potential plant bioengineering techniques have been hampered because of this key hurdle. Researchers have observed that NMs and carbon nanotubes with diameters between 3 and 50 nm may easily enter the cells of a variety of plants [108]. Small particles that may enter the phloem or xylem vessels can then translocate from one region of a plant to another, or from one organ to another. The capacity to ingress fluids from the phloem and collect NMs is surprisingly widespread throughout seeds, flowers, and fruits. The buildup of NMs in specialized organs, in addition to their toxicity to plants, raises questions regarding the safety of their use in plants for animal and human consumption [103]. 

## 9. Phytotoxicity Exhibited by Nanomaterials

There is a correlation between NM content, surface charge, size, shape, and phytotoxicity. Numerous scientific investigations have shown that NMs are poisonous to plants, with increasing toxicity at higher doses. High quantities of 25 nm AgNPs were shown to be toxic to the root cells of *Oryza sativa*, leading to cell wall disintegration and vacuole damage [103]. Concentrations of AgNPs up to 30 g/mL were found to be too high to penetrate into the root cells of *O. sativa*, but at higher concentrations, the particles caused cell disruption and toxicity [109]. The authors observed that doses of 30 g/mL promote root formation, and doses of 60 g/mL inhibit it. The findings indicate that plant damage necessitates AgNP penetration, but that at the lower penetration levels found in nature, AgNPs may benefit plants. Copper nanoparticles (CuNPs) have several applications and are employed in numerous fields. Some of these include catalysis, heat transfer, electronics, batteries, antimicrobials, and gas sensors [110]. Copper oxide nanoparticles (CuONPs) are more hazardous than copper nanoparticles (CuNPs)due to their oxidative properties. CuONPs were hypothesized to have a sizable effect on *Elodea densa* and boost photosynthesis at low concentrations, i.e., below 0.25 mg/L. A significant reduction in photosynthesis was identified at 1 mg/L; however, the impact condition varies drastically with increasing doses [111]. Other studies have also discovered phytotoxicity. The cytotoxicity of NMs might also be affected by the application techniques. Researchers have observed that NM phytotoxicity depends on more than just NM content, with structure and size also playing a role. It also depends on how much nanomaterials are being utilized. The root surface may serve as a location for NM adsorption and plant interaction, with NM absorption progressing from the root to the shoot and leaf. The pore size of a plant’s cell wall affects NM translocation. When it comes to NMs, surface charge plays a role in translocation [112]. Several studies have revealed that negatively charged NMs translocate more efficiently within plants and have less phytotoxicity than positively charged NMs. Our expanding understanding of the toxicity associated with NMs in agricultural plants is crucial to the creation of innovative agro-nanotech commodities and tools, but this understanding is still in its infancy [113]. Contradictory results have been found in current NM research in agriculture, which have focused on unexpected scenarios such as high-dose and short-term exposure and in plant species and model environments [114]. Most of these investigations have indicated that when cultured species are exposed to high concentrations of metal-based NPs, they undergo an oxidative burst. This is due to the genotoxic effects of reactive oxygen species and the fact that NPs obstruct detoxifying machinery necessary for dealing with these species [115]. As a result, the plant’s hormonal balance, growth, and secondary metabolism are frequently upset. Recent systems biology research [116] showed that metal NMs generate a universal stress response, as indicated by omics data from kidney beans, rice, wheat, rocket salad, and tobacco, and the emergence of oxidative stress components. Even if phenotypical signs of NP phytotoxicity are absent, our findings highlight the importance of performing more high-throughput research into the genetic and metabolic responses triggered by NP exposure [117]. 

## 10. Biochemical, Molecular, and Physiological Responses

The detrimental effects of NMs are complicated by a wide range of molecular, biochemical, and physiological barriers. Increased NM levels may have a deleterious effect on several plant functions, including chlorophyll content, photosynthetic rates, transpiration, the oxygen-evolving complex, and electron transport activities [118]. The expression of genes involved in photosynthesis is suppressed because NMs adhere to DNA, causing distortion and strand nicking. Genomes encoding components of the chlorophyll biosynthetic pathway and the photosystem structure are among them [119]. The NM phytotoxicity can be altered on the molecular, biochemical, and physiological levels. 

## 11. Applications of Nanomaterials in Biocontrol and Plant Growth Promotion

### 11.1. Plant Development

Nanofertilizers are an alternative to conventional fertilizers that have been shown to improve plant growth [120]. Because of their nanoscale size, nanofertilizers offer a large surface area for effective sorption. When nanofertilizers are released in a controlled manner, beneficial results like reduced toxicity and increased soil fertility can occur. Plant growth can also be stunted by a variety of abiotic factors. Oxidative stress results from the increased generation of reactive oxidative species [121]. When there is an overabundance of reactive oxidant species, genotoxicity and cytotoxicity develop, stunting plant development [122]. Nanomaterials serve a crucial purpose in protecting against abiotic stress. Antioxidant enzymes aid in plant growth by scavenging reactive oxidative species. They may also be able to generate resilience in the face of adversity [97]. Fullerenes, carbon nanotubes, and other carbon-based nanomaterials have been widely used in recent years to encourage more robust plant development because of their ability to pierce the seed coat and travel freely throughout the roots, stems, and leaves of the plant [123]. Whether or not nanoparticles can be translocated depends heavily on their size and surface charge. As compared to carbon-based nanoparticles, metal ones may accumulate within plants due to their negatively charged surfaces, demonstrating a reduced capacity for translocation [124]. The size and chemical content of nanoparticles determine their propensity to translocate within the plant, where they can stimulate growth and provide resistance to environmental stresses [125]. 

### 11.2. Nanoparticles as Biocontrol Agents

There have been many negative effects on human health and the environment as a result of the widespread usage of chemical pesticides and herbicides. Numerous nanopesticides and nanofungicides, such as those based on silver, copper, silicon dioxide, and zinc oxide, have been created to simultaneously offer high and broad-spectrum protection to plants [126]. Conventional pesticides/fungicides are highly destructive, and they have accelerated the destruction of ecosystems [127]. Nanopesticides, or nano plant protection systems, are a novel area of study that shows promise for enhancing the effectiveness of conventional pesticides in a variety of ways [128]. Some examples of nanopesticide formulations include nanoemulsions, nanocapsules (e.g., with polymers), nanoparticles with metal oxides, and nanoclays [129]. As the use of nanopesticides grows, so too are questions about governments’ capacity to appropriately analyze the risks they pose. Using nanoparticles as a vehicle, helpful bacteria that prey on plant diseases may be dispersed throughout plant tissue. Biocontrol agents, such as enzymes and antibiotics, can be delivered to plant surfaces using nanoparticles. Powdery mildew on grapevine leaves can be prevented or reduced by using gold nanoparticles [130]. Bacterial infections in plants are easy to manage with the help of silver nanoparticles. To keep plant diseases at bay, fungicides like zinc oxide nanoparticles have been utilized. Nematode control in plants is facilitated by the delivery of biocontrol agents via chitosan nanoparticles [131]. Phytopathogenic bacteria in plants have been managed with the use of iron oxide nanoparticles. Improved stability and bioavailability are two ways in which nanoparticles can be employed to enhance the effectiveness of current biocontrol drugs. Using nanoparticles, farmers can minimize specific plant pests and diseases causing collateral damage [84]. To keep plant infections at bay without resorting to harmful synthetic chemical pesticides, nanoparticles can be utilized. Being more effective, longer-lasting, and requiring fewer amounts of active ingredients are just some of the benefits of using nanotechnology in plant protection [132]. 

### 11.3. Nanoparticles as Biopesticides

The agricultural sector has seen the development of a wide variety of nanoformulations, including but not limited to nanoemulsions, nanocapsules, nanogels, and nanospheres. Nanocapsules are covered by a coating that permits them to oxidize rapidly and readily while retaining their usage. This coating made out of Nanoscale, a polymer that is safe for humans and animals. In dry areas, nanoencapsulations might have a significant impact. Because of the encapsulation, less herbicide is lost via evaporation [133]. Poly(epsilon caprolactone) nanocapsules are commonly used as a nanoformulation for enclosing atrazine. In addition, these nanocapsules can be used with environmentally safe neem oil [134]. Hexaconazole was delivered via nanocapsules manufactured and filled using an ionotropic gelation method [135]. Nanocapsules are tiny plastic encasements with a hollow inside and a protective shell. The structure is also known as a polymersome, and it consists of a bilayer or monolayer covering of an amphiphilic di- or triblock (ABA) copolymer, where A and B represent the hydrophilic and hydrophobic fragments, respectively, enclosing a water-filled cavity. It is also possible to create polymeric nanocapsules by starting with preformed polymers or by polymerizing appropriate monomers [136]. The normal size of a nano gel is around 100 nm; however, by altering its dissolvable quality and expanding its volume division, it may be kept in a three-dimensional structure with relative simplicity. In [64], myristic acid–chitosan (MA–chitosan) nanogels were prepared and subsequently loaded with *Carum copticum* essential oil (L.). The synthetic manufacture of *C. copticum* basic oil is expected to involve six known components, with thymol, p-cymene, and terpinolene being the constituents of the oil. Nanogels made from simple oils might be used to keep stored items protected from insects rather than manufactured insecticides [137]. Nanogels are naturally occurring, highly cross-connected frameworks that can be composed of a wide variety of polymers, some of which can be co-polymerized while others exist only as monomers [138]. 

Electrospun nanofibers have several potential applications, including the safe delivery of chemicals like fertilizers and pesticides. The bioactive component is evenly distributed over the polymer matrix in nanospheres, creating a unified, seamless whole [139]. Silicon nanotechnology spheres have been shown to have the potential to become significant agricultural players. There are several ways in which silicon helps plants to cope with stress. In addition to its many other benefits, silicon nanospheres have been shown to improve soil water retention and facilitate the transfer of proteins and other bioactive components [140]. 

### 11.4. Crop-Focused Nanoformulations

The advancement of nanomaterials has allowed us to more precisely and effectively combat the problems associated with conventional chemical fertilizers and pesticides. Pollution, loss of biodiversity, decreased nitrogen fixation, increased bioaccumulation of pesticides, and the introduction of new pests and illnesses into the agricultural sector are only some of the negative outcomes of the excessive usage of these chemicals [27]. Nanoformulations offer an efficient method for site-specific fertilizer application by boosting pesticide efficacy, minimizing dosage requirements, improving payload stability, and reducing run-off [141]. The term “nanoformulation” refers to the process by which active chemical compounds and other organic and inorganic substances are combined with nanoparticles, all of which must be of a nanometric size and hence cannot be viewed as a separate entity [142]. To slow down and target the distribution of agrochemicals’ active components while lessening their influence on non-target species and the ecosystem, these nanoparticles and formulations based on nanoparticles mediate a strong nanoscale system [143]. Low-dose pesticides or fertilizers may now be safely administered thanks to nanoformulations that reduce their toxicity while increasing their efficacy [144]. 

The agriculture and food sectors are investing enormous time, energy, and money into researching, designing, patenting, and producing a broad variety of functional nanoformulations to battle the pervasiveness of pests, weeds, and insects [16]. Nanoformulations of pesticides offer a number of advantages, including increased effectiveness and durability, good dispersion and wettability, the ability to biodegrade in the soil and environment, a lack of toxicity, a photogenerative nature, reduced risk of pest resistance, increased uptake by target pests, and decreased ecological contamination [145]. Nanoparticle-based pesticide formulations are attracting a lot of attention as potentially effective tools for pest management due to their ability to overcome the disadvantages of conventional pesticides. These breakthroughs may prove to be the most effective means of mitigating harmful effects on the environment over time. Pesticide nanoformulations have been linked in many studies to the development of systemic acquired resistance in plants [146]. Novel pesticide nanoformulations have been proposed and developed, including microemulsions, nanoemulsions, nanosuspensions, nanodispersions, nanoencapsulations, and even products containing purely designed nanoparticles including metals, metal oxides, nanoclays, nanospheres, nanomicelles, and nanogels. Table 1 provides a depiction of the nano-based fertilizers used for various pulse, cereal, and millet crops. 

These strategies shed light on the path toward the efficient and environmentally friendly targeted application of fungicides and insecticides in the fight against plant epidemics. In conclusion, nanoformulations may alter agricultural practices, and current research tends to produce formulations that are less harmful to the environment. Nonetheless, issues with novel formulations need to be resolved [168]. For this reason, it is essential to perform a follow-up study on nanopesticide and nanofertilizer formulations to address their potential hazards. Arabinoxylan (AXE), a polymer abundant in cellulose and hemicellulose, has been used by researchers to transform wheat bran into useful nanomaterials [169]. They promoted self-assembly into stable nanoparticles that could carry CRISPR-Cas9 DNA by altering the charge distribution of AXE. These agriculturally derived nanoparticles have the potential to distribute genes in agrochemical environments in a sustainable manner.

### 11.5. Genetic Engineering for Nanoparticle Synthesis

Increasing the synthesis of polyhydroxybutyrate (PHB), a carbon component that may be found in eco-friendly biodegradable polymers, is one of the most important roles that genetic engineering can play in the creation of nanoparticles for use in biofertilizer applications. This can be accomplished via the use of genetic engineering.

*Synechocystis* sp. PCC 6803 is a kind of cyanobacteria that has been used by researchers to explore the influence of genetic engineering and nanoparticle studies on the enhanced synthesis of PHB [170]. Because this specific strain of cyanobacteria’s genome has been analyzed in its entirety, it is a strong contender for use in nanotechnology. Techniques derived from genetic engineering have been used in an effort to ramp up the production of PHB. A PHA (polyhydroxyalkanoate)-negative mutant of *Alcaligenes eutrophus* was transformed using plasmids that included *pha* genes from both *Synechocystis* sp. PCC 6803 and *A. eutrophus*. Exogenous phaECSyn or a hybrid combination of phaECAB (phaECSyn plus phaABAe) were both components of these genes. Sugar (such as gluconate) was used as the carbon source, and the altered bacteria were able to efficiently thrive in settings devoid of nitrogen while still functioning normally. In contrast, when *Synechocystis* sp. PCC 6803 was deprived of phosphate, the strain itself generated 0.53 g/L of 3-hydroxybutyrate (3HB). Importantly, the byproducts were readily released from the cells even without the overexpression of transporters; this strain featured endogenous *phaAB* and *E. coli tesB* genes, but it lacked endogenous *phaEC* genes [151]. This allowed the byproducts to be easily released from the cells. There has been a significant amount of research conducted on the synthesis of PHB and PHA (polyhydroxyalkanoates) in both engineered and non-engineered strains of cyanobacteria as well as in other types of bacteria. There is potential for a significant amount of biomass to be generated using PHB, measured in terms of dry cell weight. Nevertheless, more studies are required to examine novel expression vectors and to increase comprehension of PHB overproduction. 

The presence of polystyrene particles, both micro and nano in size, has emerged as a major cause for worry in marine ecosystems. However, there is still a lack of clarity on the influence that they have on producers of freshwater. *S. elongatus* is a species of primary producer that lives in freshwater. For the purpose of shedding light on this topic, researchers undertook a study to explore the effects of short-term exposure to amino group-modified polystyrene nanoparticles (PS-NH2; 50 nm). The metabolite profiles and signaling pathways of the organism were the primary focuses of the investigation. According to the findings, PS-NH2 had a harmful impact on *S. elongatus*, with an EC50 value of 3.81 grammes per milliliter [152]. Through their investigation of metabolites that were not specifically targeted, the researchers were able to deduce that oxidative stress and membrane disintegration played a substantial part in the toxicity that PS-NH2 produced. In order to provide further evidence in support of these results, two modified strains of *S. elongatus* were employed. These strains indicated that glutathione metabolism was disrupted, and membrane integrity was compromised. These results add to our knowledge of the possible consequences that polystyrene nanoparticles might have on primary producers in freshwater. In the context of biofertilizer applications, they may be able to reduce the deleterious effects of polystyrene nanoparticles by making use of approaches from the field of genetic engineering. In this context, “strategies” might include changing the genetic makeup of microorganisms participating in biofertilizer production in order to increase the microorganisms’ tolerance to polystyrene nanoparticles or to promote the breakdown and decomposition of polystyrene nanoparticles in the environment. Both of these strategies are intended to improve the situation. Researchers are able to investigate ways to reduce the negative effects of polystyrene nanoparticles on the environment and create more sustainable biofertilizer production systems that are both kind to the environment and beneficial to the health of freshwater ecosystems by using the power of genetic engineering [171].

In general, approaches for genetic engineering, when combined with research on nanoparticles, provide interesting possibilities for boosting the production of PHB, which is a valuable carbon component of biodegradable polymers. These technological breakthroughs have the potential to encourage environmentally friendly farming practices and contribute to the creation of biofertilizers, both of which would be beneficial.

### 11.6. Toxicity of Nanomaterials in Food

By analyzing changes in liver and kidney function, lipid profiles, and the histology of liver and kidney tissues in rats fed on white kidney beans (WKB) fertilized with zinc oxide nanoparticles (ZnO-NPs), [172] analyzed the possible harmful consequences of ingesting these beans. Four groups of male albino rats were used in this study: a control group that received a balanced diet; a group given WKB treated with normal ZnO (nWKB); a group fed with WKB treated with 20 ppm ZnO-NPs (tWKB-1); and a group fed with WKB treated with 40 ppm ZnO-NPs (tWKB-2). According to the findings, body weight decreased in rats fed ZnONP-treated WKB, while their relative liver and spleen weights rose. Most biochemical measures showed little variations from the control group. Notably, as compared to the nWKB group, the alkaline phosphatase and aspartate transaminase activity of the tWKB-2 group were lower. These results raise concerns about possible negative physiological effects of ZnONP-treated WKB and call for more research into the safety of nanomaterials used in food production. In summary, the use of nanotechnologies in agriculture has produced useful tools, but another study’s emphasis on molybdenum oxide nanoparticles (MoO_3_NPs) raises several crucial questions [173]. Different results were seen in rats orally exposed to MoO_3_NPs and MoO_3_NPs-fertilized common beans (CB) via hematological, biochemical, and histopathological analyses. ALT, albumin, total protein, body weight change (BWC), alkaline phosphatase (ALP), lactate dehydrogenase (LDH), creatinine, creatine kinase-MB (CK-MB), thyroid-stimulating hormone (TSH), free triiodothyronine (FT3), and testosterone levels were all significantly altered in the first study after oral administration of 10 and 40 ppm MoO_3_-NPs. Similar increases were shown in BWC, total food intake (TFI), and relative kidney weight in the second trial using CB-fertilized animals. Notable changes were also seen in ALT, LDH, TSH, FT3, and testosterone levels. Notably, in both tests, the adverse effects were more evident at greater dosages of MoO_3_NPs. These results highlight the complex effects of nanomaterial exposure and emphasize the need for careful analysis when using nanotechnologies for agricultural improvement.

### 11.7. Limitations of Nanofertilizers and Methods to Improve Their Efficiency

(a) Dispersion and Stability: Poor dispersion and stability of nanofertilizers in aqueous solutions or soil often result in uneven nutrient delivery and decreased efficiency [174].
(b) Inconsistent nutrient absorption by plants using nanofertilizers may be attributed to elements such soil pH, root interactions, and nanoparticle size, which have an impact on total plant development [38].
(c) Environmental Impact: To assure environmental safety, a detailed assessment of the possible long-term impacts of nanofertilizers on soil quality, water systems, and non-target creatures is required [175].
(d) Cost: Nanofertilizers may be costly to produce, which limits their use for extensive agricultural application, especially in areas with limited resources [176].
(e) Regulatory Obstacles: Because the regulatory environment for agricultural nanotechnology is still under development, it presents difficulties for safety evaluations and approval procedures [177].

**Methods to Increase the Effectiveness of Nanofertilizers:**

(a) Particle engineering: Careful design of a nanoparticle’s characteristics, such as size, shape, and surface coating, may improve dispersion, stability, and nutrient release, hence enhancing plant absorption [178].
(b) Smart Delivery System: This can maximise nutrient availability and reduce losses by creating controlled-release nanofertilizers that release nutrients in response to plant demands or environmental circumstances [179].
(c) Nano-Enhanced Formulations: By adding nanofertilizers to traditional fertilisers or soil amendments, their overall effectiveness may be increased and their risk of side effects can be decreased [180].
(d) Organic matter or soil amendments: This improves the interactions of nanofertilizers with soil elements, enhancing nutrient retention and availability [181].
(e) Targeted Delivery: Enhancing nutrient absorption and minimizing loss may be accomplished by using nanocarriers or nanoparticles that can be directed to certain plant tissues or root zones [182].
(f) Field Studies and Monitoring: Thorough field studies, in conjunction with cutting-edge monitoring methods, may provide perceptions into the practical efficacy of nanofertilizers, assisting in their improvement [166].
(g) Environmental Assessment: Extensive research on the effects of nanofertilizers on the environment is needed to address issues with soil health, water quality, and ecosystem interactions [175].
(h) Cost-Effectiveness: By investigating the possible economic advantages of higher crop yields, as well as scalable and cost-effective manufacturing techniques, nanofertilizers may become more commercially feasible [176].

### 11.8. The Impact of Nanomaterials on the Environment, Waste Management, and the Cradle to Grave Journey

**Figure 6:**
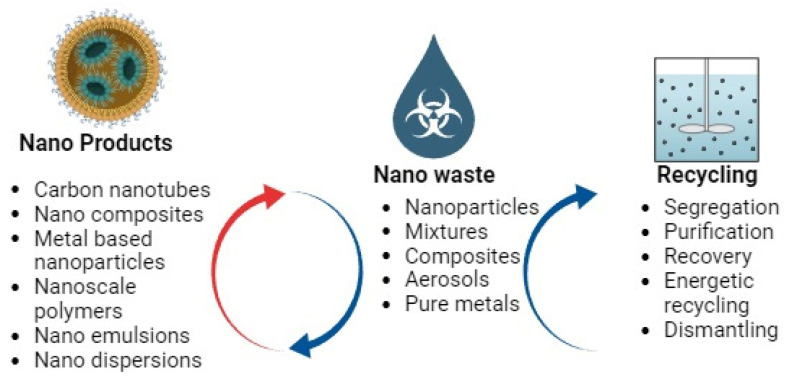
Diagram depicting nanomaterials products and their waste management. (Constructed by the authors using BioRender, Toronto, ONT, Canada).

Due to their distinctive qualities and wide range of uses in fields including electronics, health, and materials science, nanomaterials—engineered compounds at the nanoscale scale—have gained popularity. However, because to their increasing use, a thorough analysis of their life cycle, nanomaterial waste management and recycling, and possible environmental effects is required (Figure 6).

#### 11.8.1. Nanomaterials’ Cradle to Grave Journey

1. Raw Material Extraction: The extraction of certain raw materials is often necessary for the manufacturing of nanomaterials. Environmental effects of the extraction process might include habitat destruction, resource depletion, and energy use [183].
2. Production: The production of nanomaterials requires complex procedures and often consumes a lot of energy and resources. These procedures may result in waste production, pollutants, and other dangers to a worker’s occupational health [184].
3. Use: Nanomaterials are used in a variety of fields, including electronics and the medical field. Due to their small size, they may have improved qualities like better conductivity or medication delivery. However, their use may have unforeseen repercussions, demanding cautious assessment [185].
4. Disposal: Disposing of nanoparticles poses difficulties. Due to their special characteristics, nanomaterial waste may be difficult for conventional waste treatment methods to break down or neutralize. To guarantee a low influence on the environment, specialized disposal techniques must be developed [186].

#### 11.8.2. Management and Disposal of Nanomaterials Waste

Waste nanomaterials must be disposed of properly to avoid possible risks to the environment and public health. Traditional waste treatment techniques may not be enough for these materials because of their tiny size and unique features. There is continuing research into efficient disposal methods, including:

1. Controlled Landfills: Creating landfills exclusively for the disposal of nanomaterials, where containment and monitoring devices might reduce any possible leakage into the soil and groundwater [187].
2. Recycling and Reclamation: Creating methods to salvage useful parts from nanomaterial waste for reuse, obviating the necessity for fresh resource extraction [188].
3. Encapsulation and Stabilisation: Developing stabilising matrices for encapsulating nanomaterials to prevent them from dispersing into the environment [189].
4. Treatment Technologies: Researching cutting-edge treatment methods such as nanoremediation, which uses nanomaterials to clean up polluted places [185].

**Negative effects on animal and plant life include:**

According to research, nanoparticles may negatively impact plant and animal life. These outcomes comprise:

1. Impact on Soil and Plants: Nanoparticles may change the structure and makeup of soil, which can impair water retention and nutrient availability. They could also be absorbed by plants, which might have an effect on food chains and growth [190].
2. Aquatic Ecosystems: Nanomaterials may penetrate aquatic habitats, where they may have an impact on aquatic life and be bioabsorbed into the food chain. It is as yet uncertain how they may affect human health and aquatic ecosystems [191].
3. Biodiversity and Ecosystem Health: Because even small changes may upset sensitive balances, there are worries about how long-term usage of nanomaterials will affect biodiversity and ecosystem dynamics [190].

The usage of nanomaterials has the potential to significantly advance several sectors, but it is crucial to have a thorough knowledge of their life cycle, waste disposal, and possible environmental effects. To enable the safe and sustainable integration of nanomaterials into our technological environment, responsible development, thorough research, and stakeholder participation are required.

## 12. Conclusions

Nanotechnology’s unique approaches to resolving agriculture issues make it a game-changer. Our understanding of nanoparticle–plant interactions is still in its infancy. Nano-biofertilizer formulations can be helpful for sustainable agriculture development. Selection of suitable nanocarriers with no toxicity is also an important concern for effective nano-biofertilizer formulations. However, additional research is required to understand the effects of NMs on plant growth and to develop intelligent nanotechnology that can be employed to increase crop yields. Even though NMs have many positive effects on plants, phytotoxicity remains a concern. NMs have the potential to have an effect on plants at the cellular and molecular levels; thus, further study is required to understand their role in plants under normal and stressful conditions. The exact mechanism by which NMs interact with plants is unknown, but such a study might prove valuable in the future. The success of interdisciplinary collaborative techniques to overcome the gap in our understanding of the link between nanotechnology and plants is expected to be critical to the extension of the use of NMs in agriculture and, most crucially, in plant science research. 

## Figures and Tables

**Table 1:** Depiction of the nano-based fertilizers used for various pulse, cereal, and millet crops.

	Common Names	Scientific Names	Commercial Name of the Final Product	Microbes or Biomolecules	Nanocomposite	Stress Conditions	Mode of Application	Impact on Plant	Reference
Pulse Crops	1. Chickpea	*Cicer arietinum*	-	pluramin amino acid	Iron + zinc nano-fertilizers	Normal	Seed	Increase in pod numbers, grain yield, and biomass	[147]
	2. Soyabean	*Glycine max*	ZnONP	*Rhizobium japoniscum*	Zn^2+^	Normal	Seed	Increase in seed yield	[148]
	3. Mungbean	*Vigna radiata*	As-ZnO NPs	*Azospirillum*	Zn^2+^	Normal	Seed	Increase in seed germination rate and leaf area index	[149]
	4. Urdbean	*V. mungo*	IFFCO nano fertilizer	-	Nano-Zn and Nano-Cu	Normal	Seed	Increase in grain yield	[146]
	5. Dry bean	*Phaseolus vulgaris*	MN-NPs	-	ZnO, MnO_2_ and MoO_3_	Normal	Seed	Improvement in vegetative yield, flower number, and yield	[150]
	6. Lentil	*Lens culinaris*	MgO-NPs	-	MgO	Normal	Seed	Improvement in protein content	[151]
	7. Pigeonpea	*Cajanus cajan*	CuO-NP	-	CuO	Normal	Soil	Increase in total yield	[152]
	8. Cowpea	*Vigna unguiculata*	-	*Burkholderia seminalis*	nanohydroxyapatite (nHA) particles	Normal	Seed	Growth of endophytic root nodules	[100]
	9. Dry broad beans	*Vicia faba*	FeNPs	-	Fe	Normal	Seed	Increase in the production of growth-promoting phytohormones and photosynthetic pigments	[153]
	10. Dry peas	*Pisum sativum*	FA–APP @ ZnO	fulvic acid (FA) and ammonium phosphate	ZnO nanorods	Normal	Seed	Induced stronger roots, and increase in yield	[154]
	11. Peanut beans	*Arachis hypogaea*	-	-	nano-zeolite phosphorus	Normal	Seed	Increased yield of pods, and oil content	[155]
	12. Vetches	*Vicia sativa*	-	-	CuONPs, ZnONPs, MgHNPs, and MgONPs	Normal	Seed	Increased the number of bean pods	[156]
	13. Lupins	*Lupinus mutabilis*	-	-	ZnO_MnO-NPs	Normal	Seed	Increase in root size and photosynthetic pigments	[157]
Cereal crops	1. Wheat	*Triticum aestivum*	ZnO	-	zinc nitrate	Oxidative stress	Seed	Increase in catalase, peroxidase, and superoxide dismutase	[158]
	2. Barley	*Hordeum vulgare*	-	*Acinetobacter baumannii*	Nano phosphozink	Normal	Foliar spray	Improvement in the yield of Barley	[159]
	3. Oats	*Avena sativa*	-	-	Nano copper	Normal	Seed	Increase in the yield of oats	[160]
	4. Rye	*Secale cereale*	Nagro	-	Nagro	Normal	Seed	Improvement in the phytohormones	[161]
	5. Triticale	*Triticosecale*	-	*Azotobacter crocococcus, Azospirillium methylpofrom,* and *Pseudomonas putida*	Nano Fe	Normal	Seed	Increase in yield and photosynthetic pigments	[162]
	6. Maize	*Zea mays*	ZnO	-	ZnO	Normal	Seed	Improvement in growth, photosynthetic pigments, and antioxidant in maize	[163]
	7. Rice	*Oryza sativa*	-	-	nano-N (nN), nano-P (nP), nano-K (nK), and nano-NPK (nNPK)	Normal	Seed	Grain quality enhanced	[164]
Millets	1. Sorghum	*Sorghum bicolor (L.)*	-	*Azotobacter*	Nano-fertilizer	Normal	Foliar spray	Increased chlorophyll content, carotenoid	[13]
	2. Pearl Millet	*Pennisetum glaucum*	-	-	Zn and Ag Nanoparticles	Normal	Foliar spray	Caused toxicity and oxidative stress	[146]
	3. Finger Millet	*Eleusine coracana*	IFFCO nano urea	-	nano urea	Normal	Foliar spray	Overcame deficiency of nitrogen in the soil	[165]
	4. Foxtail Millet	*Setaria italica*	-	-	ZnO NPs	Normal	Foliar spray	Improved grain nutritional properties	[166]
	5. Kodo Millet	*Paspalum scrobiculatum*	-	-	Fe_2_SO_4_	Normal	Foliar spray	Increase in the grain weight and grain yield	[167]

## Data Availability

Data are available within this article.
